# Serum Is Not Necessary for Prior Pharmacological Activation of AMPK to Increase Insulin Sensitivity of Mouse Skeletal Muscle

**DOI:** 10.3390/ijms19041201

**Published:** 2018-04-15

**Authors:** Nicolas O. Jørgensen, Jørgen F. P. Wojtaszewski, Rasmus Kjøbsted

**Affiliations:** Section of Molecular Physiology, Department of Nutrition, Exercise and Sports, Faculty of Science, University of Copenhagen, DK-2100 Copenhagen, Denmark; nioj@nexs.ku.dk (N.O.J.); rasmus.kjobsted@nexs.ku.dk (R.K.)

**Keywords:** exercise, glucose uptake, AMP-activated protein kinase, TBC1D4, AS160

## Abstract

Exercise, contraction, and pharmacological activation of AMP-activated protein kinase (AMPK) by 5-aminoimidazole-4-carboxamide ribonucleotide (AICAR) have all been shown to increase muscle insulin sensitivity for glucose uptake. Intriguingly, improvements in insulin sensitivity following contraction of isolated rat and mouse skeletal muscle and prior AICAR stimulation of isolated rat skeletal muscle seem to depend on an unknown factor present in serum. One study recently questioned this requirement of a serum factor by showing serum-independency with muscle from old rats. Whether a serum factor is necessary for prior AICAR stimulation to increase insulin sensitivity of mouse skeletal muscle is not known. Therefore, we investigated the necessity of serum for this effect of AICAR in mouse skeletal muscle. We found that the ability of prior AICAR stimulation to improve insulin sensitivity of mouse skeletal muscle did not depend on the presence of serum during AICAR stimulation. Although prior AICAR stimulation did not enhance proximal insulin signaling, insulin-stimulated phosphorylation of Tre-2/BUB2/CDC16- domain family member 4 (TBC1D4) Ser711 was greater in prior AICAR-stimulated muscle compared to all other groups. These results imply that the presence of a serum factor is not necessary for prior AMPK activation by AICAR to enhance insulin sensitivity of mouse skeletal muscle.

## 1. Introduction

Skeletal muscle accounts for the vast majority of whole body glucose disposal in response to insulin [1]. Because muscle insulin resistance is a major cause of metabolic diseases such as type 2 diabetes [2], identifying molecular mechanisms involved in the regulation of muscle insulin sensitivity is central for the development of pharmacological therapies. Interestingly, exercise in the form of running and swimming, as well as contraction of isolated muscle has been shown to increase insulin sensitivity for glucose uptake in healthy and insulin resistant skeletal muscle [3,4,5,6,7,8,9,10,11]. Recently, we have provided genetic evidence to support that the insulin-sensitizing effect of exercise, contraction, and 5-aminoimidazole-4-carboxaminde ribonucleotide (AICAR) stimulation is dependent of AMP-activated protein kinase (AMPK) in skeletal muscle [11,12].

Improved muscle insulin sensitivity after contraction, AICAR stimulation, and presumably exercise further seems to depend on an unknown humoral factor present in serum [13,14]. Initial findings point towards one (or several) serum protein(s), as isolated muscle stimulated to contract in trypsin-treated serum does not exhibit enhanced insulin sensitivity [15]. Importantly, it has been shown that the origin of the serum factor is of no importance and is not specific for the individual species as serum collected from fasting and resting humans and rats promotes contraction-induced improvements in insulin sensitivity of rat skeletal muscle equally well [15]. Although much effort has been devoted to uncover the identity of the serum factor(s) [15,16,17,18], this has yet to be identified.

Evidence implies that a serum factor is necessary for improving insulin sensitivity after contraction of isolated rodent skeletal muscle [13,14,15,16,19] as well as after AICAR stimulation of rat skeletal muscle [14]. One study recently questioned this requirement of a serum factor by demonstrating improved insulin sensitivity after prior AICAR stimulation of isolated skeletal muscle from old rats in the absence of serum [20]. Therefore, we investigated whether the effect of AICAR on insulin sensitivity for glucose uptake in isolated mouse extensor digitorum longus (EDL) was dependent on the presence of serum, as this is currently unknown. Additionally, we examined AMPK and insulin signaling in collected muscle samples that may support the molecular and mechanistic signature of AICAR-induced improvements of muscle insulin sensitivity. 

## 2. Results

### 2.1. Acute Serum Stimulation Does Not Affect Basal or AICAR-Stimulated Glucose Uptake in Mouse Skeletal Muscle

**Figure 1:**
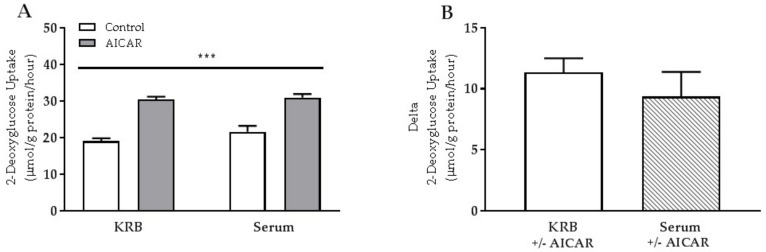
Acute serum- and 5-aminoimidazole-4-carboxamide ribonucleotide (AICAR)-stimulated glucose uptake in isolated mouse skeletal muscle. (**A**) 2-deoxyglucose (2-DG) uptake in isolated extensor digitorum longus (EDL) muscle from C57Bl/6 mice in response to 50 min of serum and/or AICAR stimulation; (**B**) Delta 2-DG uptake (AICAR minus control) in Krebs Ringer buffer (KRB) and serum-stimulated muscles. Data were analyzed by a two-way repeated-measures analysis of variance (ANOVA) and a Student’s *t*-test, respectively. *** *p* < 0.001 indicates main effect of AICAR. Values are means ± SEM. *n* = 8 in all groups.

Glucose uptake increased in EDL muscle in response to acute AICAR stimulation (Figure 1A). Neither basal nor AICAR-stimulated glucose uptake was affected by the presence of serum (Figure 1A,B).

**Figure 2:**
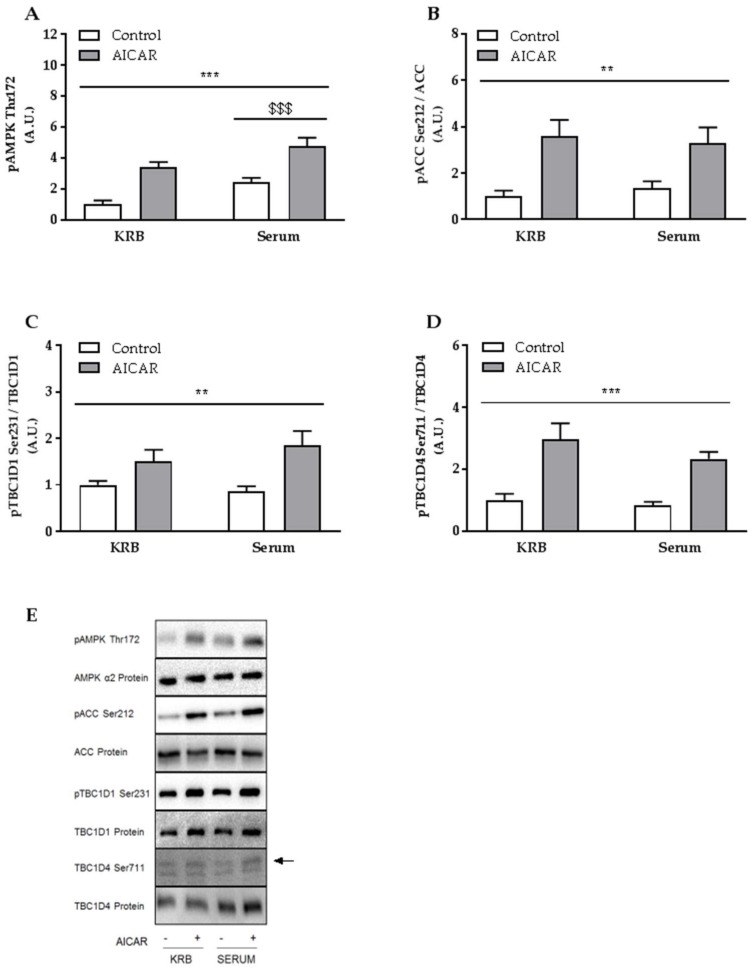
AMP-activated protein kinase (AMPK) signaling in isolated mouse skeletal muscle following acute serum and AICAR stimulation. (**A**) Phosphorylation of AMPK Thr172; (**B**) Acetyl-CoA carboxylase (ACC) Ser212; (**C**) Tre-2/BUB2/CDC16-domain family member 1 (TBC1D1) Ser231; and (**D**) Tre-2/BUB2/CDC16-domain family member 4 (TBC1D4) Ser711, in isolated EDL muscle from C57Bl/6 mice in response to 50 min of serum and/or AICAR stimulation; (**E**) Representative immunoblots. Data were analyzed by a two-way repeated-measures ANOVA. *** *p* < 0.001 and ** *p* < 0.01 indicate main effect of AICAR. ^$$$^ *p* < 0.001 indicates main effect of serum. Values are means ± SEM. *n* = 8 in all groups. A.U., arbitrary units.

Alongside the increase in glucose uptake, acute AICAR stimulation increased phosphorylation of AMPK Thr172, acetyl-CoA carboxylase (ACC) Ser212, Tre-2/BUB2/CDC16-domain family member 1 (TBC1D1) Ser231, and Tre-2/BUB2/CDC16-domain family member 4 (TBC1D4) Ser711 compared to control muscles (Figure 2A–D). Of these phosphorylation sites, only phosphorylation of AMPK Thr172 increased in skeletal muscle when incubated in serum (Figure 2A) compared to the standard serum-free incubation buffer. As downstream targets of AMPK was unaffected by the presence of serum, this may indicate that serum does not affect AMPK activity in incubated skeletal muscle. Besides a small decrease of AMPKα2 protein content in serum-incubated muscles, total protein abundance of the measured proteins was not affected by serum and AICAR stimulation.

### 2.2. The Absence of Serum Does Not Influence the Ability of Prior AICAR Stimulation to Increase Mouse Muscle Insulin Sensitivity

**Figure 3:**
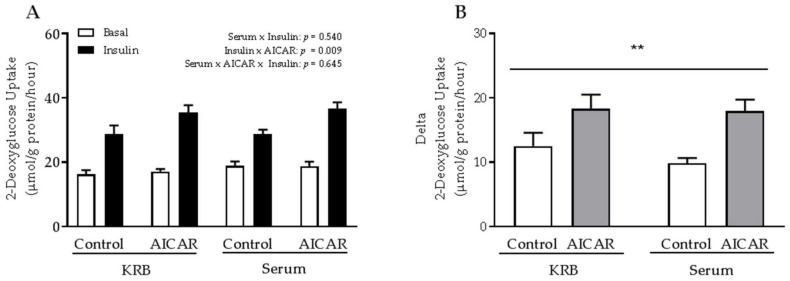
Enhanced insulin sensitivity after prior AICAR stimulation in isolated mouse skeletal muscle incubated in the absence or presence of serum. (**A**) Basal and submaximal insulin-stimulated 2-DG uptake in isolated EDL muscle from C57Bl/6 mice 6 h after prior AICAR stimulation in KRB or serum. (**B**) Delta 2-DG uptake (insulin minus basal) in prior control and AICAR-stimulated muscles. Data were analyzed by a three-way repeated-measures ANOVA and a two-way ANOVA, respectively. Possible interactions between groups are indicated in the figure. ** *p* < 0.01 indicates main effect of AICAR. Values are means ± SEM. *n* = 4–6 and *n* = 8–12 in serum and KRB group, respectively.

As acute AICAR stimulation increased glucose uptake and AMPK-related downstream signaling similarly in EDL muscle incubated in the presence or absence of serum, we tested whether serum was in fact necessary for prior AICAR stimulation to increase muscle insulin sensitivity. We found that insulin sensitivity was increased 6 h after prior AICAR stimulation in mouse EDL muscle regardless of whether or not serum was present during AICAR stimulation (Figure 3A). Thus, the incremental increase in insulin-stimulated glucose uptake was significantly higher in prior AICAR-stimulated muscle independent of serum presence (Figure 3B).

### 2.3. Increased Muscle Insulin Sensitivity Coincides with Elevated AMPK Signaling

**Figure 4:**
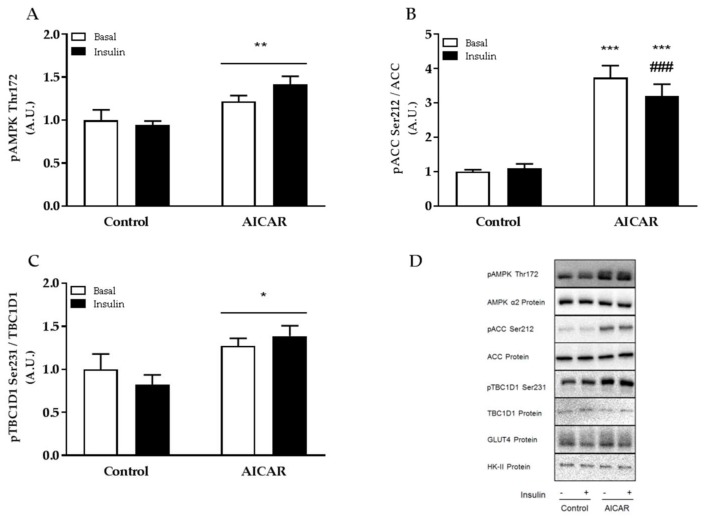
AMPK signaling in isolated mouse skeletal muscle after prior AICAR stimulation in serum-free incubation buffer. (**A**) Phosphorylation of AMPK Thr172; (**B**) ACC Ser212; and (**C**) TBC1D1 Ser231 in isolated EDL muscle from C57Bl/6 mice 6 h after prior AICAR stimulation in KRB; (**D**) Representative immunoblots. Data were analyzed by a two-way repeated-measures ANOVA. ** *p* < 0.01 and * *p* < 0.05 indicate main effect of AICAR. *** *p* < 0.001 indicates effect of AICAR within group. ^###^ *p* < 0.001 indicates effect of insulin within AICAR. Values are means ± SEM. *n* = 8 and *n* = 12 in control and AICAR group, respectively. A.U., arbitrary units.

Since we have previously reported intracellular signaling in prior serum- and AICAR-stimulated mouse EDL muscle [12], we decided to evaluate intracellular signaling only in EDL muscle incubated without serum. Previously we have shown that AMPK signaling is elevated in isolated muscle 6 h into recovery from acute AICAR and serum stimulation [12]. Concomitantly, we found that phosphorylation of AMPK Thr172, ACC Ser212, and TBC1D1 Ser231 was also increased 6 h after prior AICAR stimulation in muscle incubated without serum (Figure 4A–C). No change in total protein expression of AMPKα2, ACC, TBC1D1, Glucose transporter 4 (GLUT4), and Hexokinase II (HK-II) was found 6 h into recovery from acute AICAR stimulation.

### 2.4. Insulin-Stimulated Phosphorylation of Akt Thr308 and Ser473 Is Not Affected by Prior AICAR Stimulation

**Figure 5:**
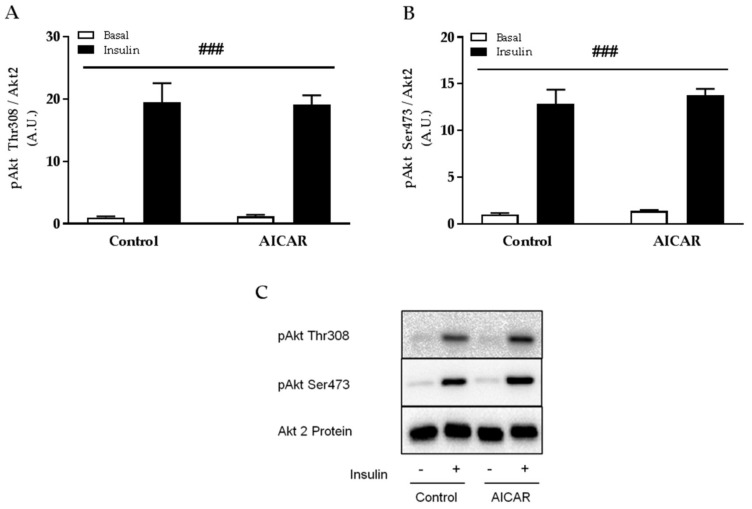
Akt signaling in isolated mouse skeletal muscle after prior AICAR stimulation in serum-free incubation buffer. Insulin-stimulated phosphorylation of (**A**) Akt Thr308 and (**B**) Ser473 in isolated EDL muscle from C57Bl/6 mice 6 h after prior AICAR stimulation in KRB. (**C**) Representative immunoblots. Data were analyzed by a two-way repeated-measures ANOVA. ^###^ *p* < 0.001 indicates main effect of insulin. Values are means ± SEM. *n* = 8 and *n* = 11 in control and AICAR group, respectively. A.U., arbitrary units.

Several observations indicate that improvements in muscle insulin sensitivity following exercise, contraction, and AICAR stimulation occur in the absence of elevated proximal insulin signaling [7,11,12,14,21,22,23]. In line, we found that submaximal insulin-stimulated phosphorylation of Akt Thr308 and Ser473 was similar between control and prior AICAR-stimulated muscles incubated without serum (Figure 5A,B). No change in total protein expression of Akt2 was found 6 h into recovery from acute AICAR stimulation.

### 2.5. Insulin-Stimulated Phosphorylation of TBC1D4 Ser711 Is Elevated in Prior AICAR-Stimulated Muscle

**Figure 6:**
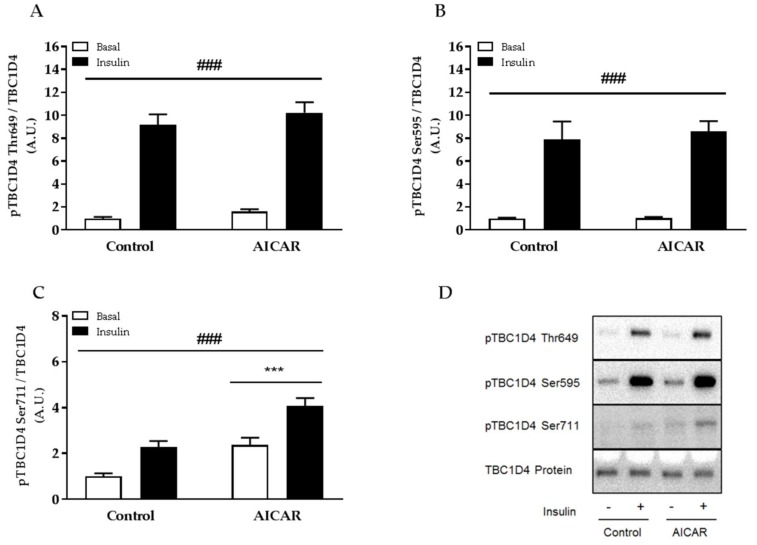
TBC1D4 signaling in isolated mouse skeletal muscle after prior AICAR stimulation in serum-free incubation buffer. Insulin-stimulated phosphorylation of (**A**) TBC1D4 Thr649; (**B**) Ser595; and (**C**) Ser711 in isolated EDL muscle from C57Bl/6 mice 6 h after prior AICAR stimulation in KRB; (**D**) Representative immunoblots. Data were analyzed by a two-way repeated-measures ANOVA. ^###^ *p* < 0.001 indicates main effect of insulin. *** *p* < 0.001 indicates main effect of AICAR. Values are means ± SEM. *n* = 8 and *n* = 12 in control and AICAR group, respectively. A.U., arbitrary units.

Phosphorylation of TBC1D4 has been shown to be important for insulin-stimulated glucose uptake in skeletal muscle [24,25] and evidence suggests that TBC1D4 may relay improvements in insulin sensitivity of muscle previously stimulated with AICAR and serum [12]. We found that insulin-stimulated phosphorylation of TBC1D4 Ser595 and Thr649 was similar between control and prior AICAR-stimulated muscles incubated without serum (Figure 6A,B). In contrast, phosphorylation of AMPK downstream target TBC1D4 Ser711 was significantly higher in prior AICAR- and insulin-stimulated muscle compared to all other groups (Figure 6C), signifying a potential role of TBC1D4 Ser711 for regulating muscle insulin sensitivity. No change in total protein expression of TBC1D4 was found 6 h into recovery from acute AICAR stimulation.

## 3. Discussion

Here, we demonstrate that improved insulin sensitivity after prior AICAR stimulation of isolated mouse skeletal muscle does not require the presence of serum. Additionally, we show that the insulin-sensitizing effect of AICAR occurs independently of elevated proximal insulin signaling but coincides with elevated insulin-stimulated phosphorylation of TBC1D4 Ser711, a known downstream target of AMPK. Thus, our data suggest that a serum factor is not needed for prior pharmacological activation of AMPK to enhance insulin sensitivity of mouse skeletal muscle in contrast to previous assumptions [12].

It has previously been suggested that the presence of a serum factor is necessary for improved insulin sensitivity after prior AICAR stimulation of rat epitrochlearis muscle [14]. The data presented herein oppose the findings by Fisher et al. [14], though the muscles studied differed with regards to type, species, and gender. However, a recent study reported that prior AICAR stimulation improved insulin sensitivity of rat epitrochlearis muscle in the absence of serum [20], emphasizing that the discrepancies observed between the present study and that of Fisher et al. [14] are not due to differences in muscle type or species. Since Oki et al. [20] and Fisher et al. [14] investigated muscle from old (24 months-old) and young (likely ~6 weeks-old) rats, respectively, the observed difference could be due to an effect of age somehow affecting the necessity of a serum factor to mediate improvements in muscle insulin sensitivity after prior AICAR stimulation. However, the mice used in the present study were young, suggesting that, at least in mice, the ability for pharmacological AMPK activation to increase skeletal muscle insulin sensitivity in the absence of serum is not restricted to aged muscle.

Since we investigated muscle from female mice in the present study, data presented here and in the study by Fisher et al. [14], where a serum factor was found necessary for AICAR-induced improvement of insulin sensitivity in muscle from young male rats, suggests that a gender difference may be responsible for the observed discrepancy. Whether a serum factor is indeed needed for prior AICAR stimulation to improve insulin sensitivity of young male mouse muscle is not known at present. Interestingly though, in a recent study, serum was found necessary for prior contraction to improve insulin sensitivity of isolated skeletal muscle from young male mice [19]. As such, we cannot exclude that gender-related differences in skeletal muscle may exist and therefore influence whether or not a serum factor has to be present for AICAR to improve muscle insulin sensitivity.

We found that acute serum stimulation of isolated mouse muscle did not affect glucose uptake or phosphorylation of TBC1D4. This is in contrast to another study showing that acute serum stimulation increases glucose uptake in isolated rat skeletal muscle as well as phosphorylation of Akt and TBC1D4 [23]. These findings were likely due to the presence of insulin in serum, as the authors reported a similar increase in glucose uptake and phosphorylation of TBC1D4 when incubating rat muscle in serum-free buffer with an insulin concentration equivalent to that found in the used serum [23]. We speculate that the inconsistency between this and the aforementioned study with regards to the acute effects of serum stimulation may relate to the use of serum from different species. Thus, although serum was obtained from healthy male rats [23] and humans (the present study) in the fasted and rested condition, fasting insulin concentrations in rat serum are typically twice as high of that found in human serum [26,27]. This may be the cause of the observed differences in glucose uptake and cellular signaling between the two studies. 

Several studies have reported that the increase in muscle insulin sensitivity after exercise, contraction, and AICAR stimulation occurs independently of enhanced proximal insulin signaling (e.g., from insulin binding to Akt activity) [7,11,12,14,21,22,23]. In line, we found that insulin-stimulated phosphorylation of Akt Thr308 and Ser473 was similar between control and prior AICAR-stimulated muscles although glucose uptake was not.

We have previously reported that AMPK downstream signaling is increased in isolated muscle 6 h after prior AICAR and serum stimulation [12]. Furthermore, this increase seems to depend on a persistent increase in AMPK α2β2γ3 activity, which likely regulates muscle insulin sensitivity [12]. In prior AICAR- but non-serum-stimulated muscle, we also found a persistent increase in phosphorylation of AMPK Thr172 and downstream targets ACC Ser212 and TBC1D1 Ser231 indicating that prior AICAR stimulation improves insulin sensitivity similarly in serum and non-serum treated muscles. Interestingly, elevated phosphorylation of AMPK Thr172, ACC Ser212 and TBC1D1 Ser231 is not found in insulin-sensitized rodent skeletal muscle after prior contraction [11,23] even though activity of the AMPK α2β2γ3 complex is increased [11]. Thus, despite that differences in phosphorylation of AMPK targets are observed between prior contracted and AICAR-stimulated skeletal muscle, increased activity of the AMPK α2β2γ3 complex is found during both conditions supporting the notion that AICAR and contraction improve muscle insulin sensitivity via the AMPK α2β2γ3 complex. 

TBC1D4 is a Rab GTPase-activating protein involved in the regulation of GLUT4 trafficking [28]. In skeletal muscle, TBC1D4 is phosphorylated by Akt, which seems important for increasing glucose uptake in response to insulin [24,25,29]. TBC1D4 is also targeted at Ser711 by AMPK during exercise, contraction, and acute AICAR stimulation [11,30]. Importantly, phosphorylation of TBC1D4 Ser711 seems to be regulated directly by the AMPK α2β2γ3 complex [11] but it does not seem to affect muscle glucose uptake per se [30]. Several findings have pointed towards a role of TBC1D4 in regulating muscle insulin sensitivity given its function as a point of convergence for exercise (AMPK) and insulin (Akt) signaling. Indeed, recent evidence from our muscle-specific AMPK transgenic mouse models supports the notion of an AMPK-TBC1D4 signaling axis involved in the regulation of muscle insulin sensitivity as both improvements in insulin-stimulated glucose uptake and phosphorylation of TBC1D4 Ser711 are abrogated in AMPK-deficient muscle after prior in situ contraction as well as prior AICAR stimulation of serum-incubated muscle [11,12]. In accordance, we found that insulin-stimulated phosphorylation of TBC1D4 was increased at Ser711 in prior AICAR-stimulated muscle concomitant with enhanced insulin sensitivity.

Taken together, improved insulin sensitivity of mouse skeletal muscle after prior pharmacological activation of AMPK by AICAR does not require the presence of a serum factor. This is in contrast to findings in prior contracted and AICAR-stimulated skeletal muscle from young male rats in which one (or several) unknown serum factor(s) seems important to enhance insulin sensitivity [13,14,15,16,23]. Moreover, considerable evidence points toward an important role of elevated phosphorylation of TBC1D4 Ser711 for enhancing muscle insulin sensitivity after AMPK activating stimuli, signifying the importance of the upstream regulator AMPK.

## 4. Materials and Methods

### 4.1. Animals

All animal experiments were approved by the Danish Animal Experiments Inspectorate (#2014-15-2934-01037, approved 4 March 2014) and complied with the EU convention for the protection of vertebra used for scientific purposes (Council of Europe, Treaty 123/170, Strassbourg, France, 1985/1998). Animals used in this study were C57Bl/6J female mice from Taconic (Ejby, Denmark). Young mice (19.9 ± 1.9 g [means ± SD]) were maintained on a 12:12 hour light-dark cycle with free access to standard rodent chow and water. Serum was obtained from a healthy man (Body Mass Index: 24.1 kg/m^2^, 34 years of age) in the overnight fasted and rested state (blood glucose concentration = 5.4 mmol/L). The serum was collected by antecubital venous catheter and kept frozen at −26 °C until used and was not refrozen for later use. Collection of human serum was approved by the Ethics Committee of Copenhagen (#H-3-2012-140, approved 29 November 2012) and complied with the ethical guidelines of the Declaration of Helsinki II. Informed consent was obtained from the serum donor before entering the study.

### 4.2. Muscle Incubations

Fed animals were anesthetized by an intraperitoneal injection of Pentobarbital (10 mg/100 g body weight) before EDL muscles were isolated and suspended in incubation chambers containing Krebs Ringer buffer (KRB) as previously described [12]. In short, EDL muscles were incubated for 50 min in the absence or presence of 1 mmol/L AICAR (Toronto Research Chemicals, Toronto, ON, Canada) in KRB or 100% human serum. Subsequent to AICAR stimulation, muscles were allowed to recover for 6 h in KRB supplemented with 5 mmol/L of D-glucose after which they were incubated in KRB with or without a submaximal insulin concentration (100 μU/mL, 30 min). 2-deoxyglucose (2-DG) uptake was measured during the last 10 min of the 30 min stimulation period by adding 1 mmol/L [^3^H]2-DG (0.028 MBq/mL) and 7 mmol/L [^14^C]mannitol (0.0083 MBq/mL) to the incubation medium. For glucose uptake measurements in response to acute AICAR and serum stimulation, EDL muscles were incubated in KRB or 100% human serum for 50 min with or without 1 mmol/L AICAR. Following stimulation all muscles were washed in KRB for 1 min before 2-DG uptake was measured during a 10 min incubation period. For measurements of acute AICAR-stimulated muscle glucose uptake, AICAR was present in the incubation medium throughout the entire incubation period. For all incubations, 2-DG uptake was determined as previously described [11]. 

### 4.3. Muscle Processing, Sodium Dodecyl Sulfate Polyacrylamide Gel Electrophoresis (SDS-PAGE), and Western Blot Analyses

Muscles were homogenized as previously described [12] and lysates were collected and frozen in liquid nitrogen for subsequent analyses. The bicinchoninic acid method was used to determine total protein abundance in muscle lysates. Lysates were boiled in Laemmli buffer and subjected to SDS-PAGE and immunoblotting as previously described [12].

### 4.4. Antibodies

Primary antibodies against Akt2 (#3063), pAkt-Ser473 (#9271), pAkt-Thr308 (#9275), pAMPKα-Thr172 (#2531), pACC-Ser79/212 (#3661), pTBC1D4-Ser588 (mouse: Ser595) (#8730), and pTBC1D4-Thr642 (mouse: Thr649) (#8881) were from Cell Signaling Technology (Danvers, MA, USA) Antibody against pTBC1D1-Ser231 (#NRG-1848963) was from Millipore (Burlington, MA, USA), AMPKα2 antibody (#SC-19131) and Hexokinase II were from Santa Cruz (Dallas, TX, USA)(#SC-6521) while GLUT4 antibody (#PA1-1065) was from Thermo Fisher Scientific (Waltham, MA, USA). ACC protein was detected using horseradish peroxidase-conjugated streptavidin from Dako (Glostrup, Denmark), (#P0397). TBC1D1 and TBC1D4 protein as well as phosphorylation of TBC1D4-Ser711 were detected using antibodies as previously described [31,30]. 

### 4.5. Statistics

Data are presented as the means ± SEM unless stated otherwise. Results on cellular signaling are presented in figures as relative to the basal or control group levels within the given experiment. An unpaired Student’s *t*-test (Figure 1B) as well as three-way (Figure 3A) and two-way (remaining figures) ANOVA with and without repeated measures were used to assess statistical differences. The Student–Newman–Keuls test was used for post hoc testing. Main effects are indicated with lines comprising the affected groups and symbols in Figure 4B represent post hoc test corrected *p*-values. *, #, and $ indicate effects of AICAR, insulin, and serum, respectively. Statistical significance was defined as *p* < 0.05.

## Abbreviations

2-DG	2-deoxyglucose
ACC	Acetyl-CoA carboxylase
AICAR	5-aminoimidazole-4-carboxamide ribonucleotide
AMPK	AMP-activated protein kinase
ANOVA	Analysis of variance
A.U.	Arbitrary units
EDL	Extensor digitorum longus
GLUT4	Glucose transporter 4
HK-II	Hexokinase II
KRB	Krebs Ringer buffer
SDS-PAGE	Sodium dodecyl sulfate polyacrylamide gel electrophoresis
TBC1D1	Tre-2/BUB2/CDC16-domain family member 1
TBC1D4	Tre-2/BUB2/CDC16-domain family member 4
